# Corneal confocal microscopy detects corneal nerve damage and increased dendritic cells in Fabry disease

**DOI:** 10.1038/s41598-018-30688-z

**Published:** 2018-08-16

**Authors:** Gulfidan Bitirgen, Kultigin Turkmen, Rayaz A. Malik, Ahmet Ozkagnici, Nazmi Zengin

**Affiliations:** 1 Department of Ophthalmology, Necmettin Erbakan University Meram Faculty of Medicine, Konya, Turkey; 2 Division of Nephrology, Department of Internal Medicine, Necmettin Erbakan University Meram Faculty of Medicine, Konya, Turkey; 3 Weill Cornell Medicine-Qatar, Education City, Doha, Qatar; 4 Central Manchester University Teaching Hospitals Foundation Trust and Division of Cardiovascular Sciences, University of Manchester, Manchester, UK

## Abstract

Fabry disease is characterised by neuropathic pain and accelerated vascular disease. This study evaluates the utility of corneal confocal microscopy (CCM) to non-invasively quantify corneal nerve and endothelial cell morphology and dendritic cell (DC) density in relation to disease severity in subjects with Fabry disease. Seventeen consecutive participants with Fabry disease and 17 healthy control subjects were included in this cross-sectional study. Fabry disease severity was measured using the Mainz Severity Score Index (MSSI). Central corneal sensitivity was assessed with a contact corneal esthesiometer. There was a significant reduction in the corneal sensitivity (5.75 [5.25–6.00] vs. 6.00 [6.00-6.00] cm, *P* = 0.014), nerve fiber density (NFD) (26.4 ± 10.1 vs. 33.7 ± 7.9 fibers/mm^2^, *P* = 0.025) and nerve fiber length (NFL) (15.9 ± 3.4 vs. 19.5 ± 4.4 mm/mm^2^, *P* = 0.012) and an increase in DC density (38.3 [17.5–97.3] vs. 13.5 [0–29.4] cells/mm^2^, *P* = 0.004) in subjects with Fabry disease compared to the healthy control subjects. The total MSSI score correlated with NFD (ρ = −0.686; *P* = 0.006), NFL (ρ = −0.692; *P* = 0.006), endothelial cell density (ρ = −0.511; *P* = 0.036), endothelial cell area (ρ = 0.514; *P* = 0.036) and α-galactosidase A enzyme activity (ρ = −0.723; *P* = 0.008). This study demonstrates reduced corneal sensitivity, corneal nerve fiber damage and increased DCs in subjects with Fabry disease.

## Introduction

Fabry disease is a rare X-linked disorder due to a deficiency or absence of the lysosomal enzyme α-galactosidase A, which results in the accumulation of the sphingolipid degradation product globotriaosylceramide^1^. The prevalence of Fabry disease has been reported as 1/476,000 in normal population and 0.95% in chronic kidney disease patients^2,3^. A common presenting symptom of Fabry disease is neuropathic pain, which usually develops during childhood^4,5^. Histopathological studies have revealed that glycolipid accumulation in the dorsal root ganglion with axonal degeneration of the small nerve fibers may cause neuropathic pain^6,7^. Electrophysiological studies, quantitative sensory testing, sural nerve and skin biopsy studies have confirmed neuropathy in subjects with Fabry disease^8–10^.

We have previously demonstrated the clinical utility of corneal confocal microscopy (CCM) as an imaging biomarker that quantifies small nerve fiber damage in diabetic neuropathy, idiopathic small fiber neuropathy, Charcot-Marie-Tooth disease type 1 A, chemotherapy induced peripheral neuropathy, sarcoid neuropathy and HIV neuropathy^11–16^. More recently CCM has also shown corneal nerve degeneration in Parkinsons disease, multiple sclerosis and stroke^17–20^. CCM has been used to detect increased Langerhans cell and dendritic cell (DC) density in keratoconus, early diabetic neuropathy, chronic inflammatory demyelinating polyneuropathy and multiple sclerosis^18,21–24^.

With regard to Fabry disease, we have previously shown a reduction in corneal sensation and a loss of corneal nerve fibers, which was related to the severity of clinical neuropathy in hemizygous males^25^. However this study was undertaken using a Tomey corneal confocal microscope which is a first generation device with limited resolution and our study only assessed corneal nerve morphology.

Accelerated vascular disease with progressive renal failure, cardiomyopathy and occlusive cerebro-vascular events are associated with early mortality in subjects with Fabry disease^26^. Whilst some clinical and experimental studies have shown that accumulation of globotriaosylceramide in endothelial cells may contribute to vascular dysfunction^27,28^, others have not^29^. Additionally, activation of innate immunity via dendritic cells has been associated with renal and cardiac inflammation in Fabry disease^30–32^.

In the current study we have undertaken CCM to quantify corneal nerve and endothelial cell morphology and DC density to explore underlying mechanisms and to establish whether CCM could contribute to the development of imaging biomarkers for tissue damage in subjects with Fabry disease.

## Results

Seventeen subjects with Fabry disease and 17 age- and sex-matched healthy control participants were enrolled in the study. The baseline characteristics of the subjects with Fabry disease and healthy controls are summarized in Table 1. The mean ages of the subjects with Fabry disease and healthy control participants did not differ (34.6 ± 13.5 vs. 34.5 ± 13.2 years, *P* = 0.980). Eleven of the 17 subjects (64.7%) were receiving enzyme replacement therapy (ERT), biweekly infusions of agalsidase alpha (Replagal^®^; Shire Human Genetic Therapies AB, Lund, Sweden). The mean period of ERT use was 12.4 ± 5.7 months (range 5–18 months). Study subjects with Fabry disease were the members of 3 different families. Thirteen participants had c.100 A > C (p.N34H) mutation, 3 participants had c.160 C > T (p.Leu54Phe) mutation and one participant had c.1072_1074delGAG (p.358delE) mutation. The average values of α-galactosidase A enzyme activity were 1.05 ± 0.51 µmol/L/h in males and 2.03 ± 0.63 µmol/L/h in females.

**Table 1:** Baseline characteristics of the study participants.

	Control group (n = 17)	Fabry disease (n = 17)	Males (n = 10)	Females (n = 7)
		Total		
Age (years)	34.5 ± 13.2	34.6 ± 13.5	30.9 ± 11.1	40.0 ± 15.6
Gender (M/F)	10/7	10/7	—	—
MSSI	—	23.5 ± 13.0	30.0 ± 13.4	14.3 ± 3.7
MSSI – Neurological score	—	8.4 ± 4.2	10.2 ± 4.4	5.9 ± 2.3
α-Galactosidase A enzyme activity (µmol/L/h)	—	1.42 ± 0.73	1.05 ± 0.51	2.03 ± 0.63
Duration of ERT use (months)	—	12.4 ± 5.7	13.4 ± 5.8	7.5 ± 2.1

ERT, enzyme replacement therapy; MSSI: Mainz Severity Score Index. Data are mean ± SD.

### Corneal sensitivity and nerve morphology

**Figure 1:**
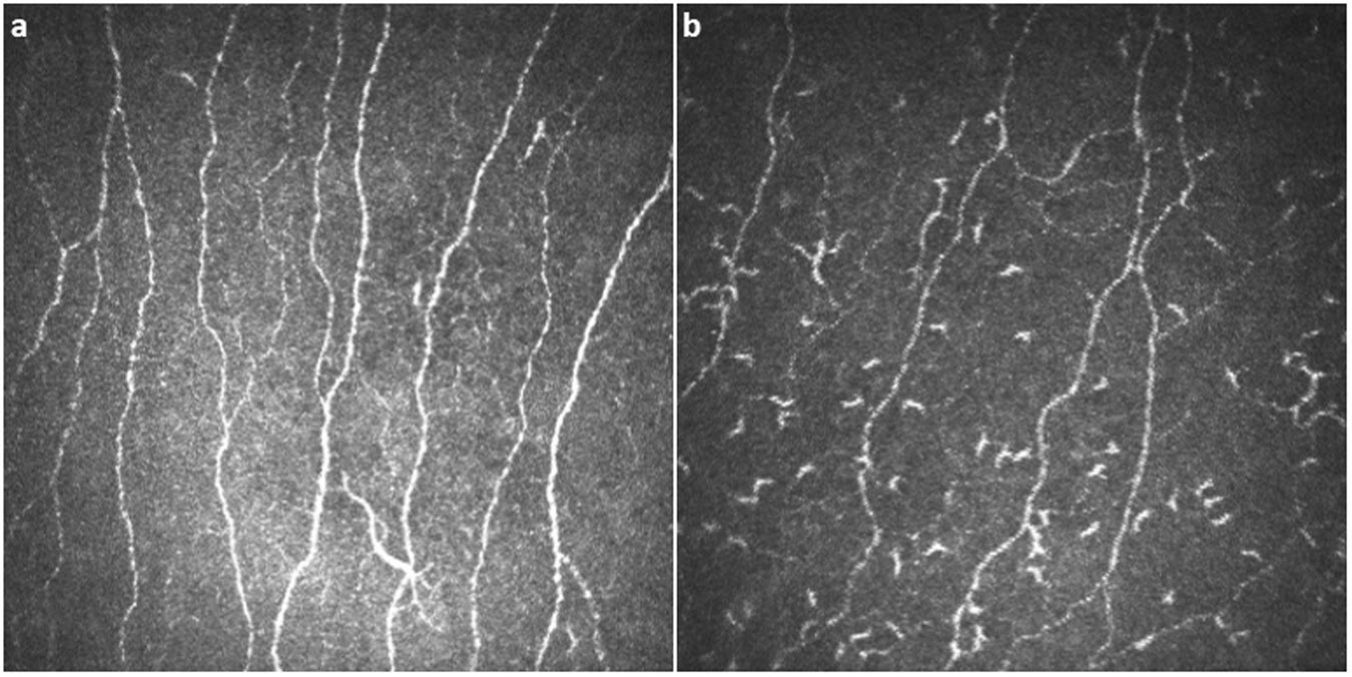
Corneal confocal microscopic images of the central cornea in a healthy control subject (**a**) and a subject with Fabry disease (**b**), showing reduced nerve fibers and increased dendritic cells.

**Figure 2:**
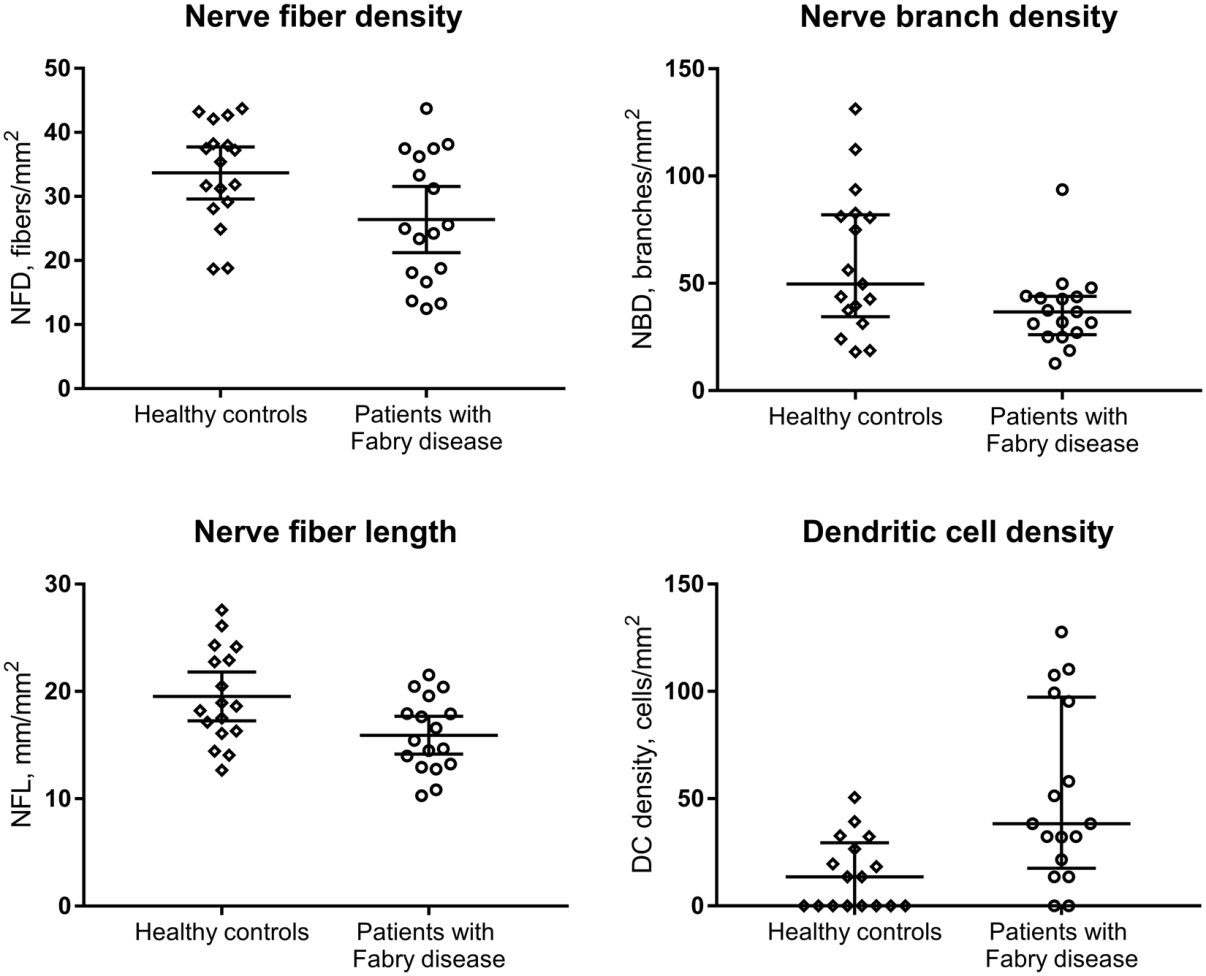
Corneal subepithelial nerve parameters and dendritic cell density in healthy control participants and subjects with Fabry disease, showing decreased NFD (*P* = 0.025) and NFL (*P* = 0.012) and increased DC density (*P* = 0.004), with no significant difference in NBD (*P* = 0.062).

Representative CCM images of the central cornea in a healthy subject and a participant with Fabry disease, demonstrating reduced nerve fibers and increased DCs are shown in Fig. 1. There were significant reductions in the central corneal mechanical thresholds (5.75 [5.25–6.00] vs. 6.00 [6.00 – 6.00] cm, *P* = 0.014), NFD (26.4 ± 10.1 vs. 33.7 ± 7.9 fibers/mm^2^, *P* = 0.025) and NFL (15.9 ± 3.4 vs. 19.5 ± 4.4 mm/mm^2^, *P* = 0.012) in participants with Fabry disease compared to control subjects (Table 2, Fig. 2). There were no significant differences in corneal sensitivity and confocal microscopic measures between participants with the p.N34H mutation and p.Leu54Phe mutation (Supplementary Table S1).

**Table 2:** Central corneal sensitivity, corneal nerve fiber parameters and dendritic cell density in subjects with Fabry disease and healthy control group.

	Control group (n = 17)	Fabry disease (n = 17)	*P* value
Central corneal sensitivity (cm, median [IQR])	6.00 [6.00 – 6.00]	5.75 [5.25–6.00]	0.014^a^
Nerve fiber density (fibers/mm^2^, mean ± SD)	33.7 ± 7.9	26.4 ± 10.1	0.025^b^
Nerve branch density (branches/mm^2^, median [IQR])	49.7 [34.4–81.9]	36.7 [26.1–43.9]	0.062^a^
Nerve fiber length (mm/mm^2^, mean ± SD)	19.5 ± 4.4	15.9 ± 3.4	0.012^b^
Dendritic cell density (cells/mm^2^, median [IQR])	13.5 [0–29.4]	38.3 [17.5–97.3]	0.004^a^

^a^Mann-Whitney test. ^b^Independent samples t-test.

### Corneal endothelial cells

Corneal endothelial cell density (3220.1 ± 196.5 vs. 3091.1 ± 189.7 cells/mm^2^, *P* = 0.060), cell area (250.6 ± 17.2 vs. 261.9 ± 17.7 µm^2^, *P* = 0.067), cell perimeter (57.3 ± 1.9 vs. 58.7 ± 2.2 µm, *P* = 0.054), pleomorphism (36.8 ± 4.0 vs. 35.3 ± 5.4%, *P* = 0.376) and polymegathism (46.3 ± 3.4 vs. 48.7 ± 4.5%, *P* = 0.086) did not differ signficantly between subjects with Fabry disease and control subjects (Table 3).

**Table 3:** Corneal endothelial cell parameters in subjects with Fabry disease and healthy control group.

	Control group (n = 17)	Fabry disease (n = 17)	*P* value^a^
Endothelial cell density (cells/mm^2^, mean ± SD)	3091.1 ± 189.7	3220.1 ± 196.5	0.060
Endothelial cell area (µm^2^, mean ± SD)	261.9 ± 17.7	250.6 ± 17.2	0.067
Endothelial cell perimeter (µm, mean ± SD)	58.7 ± 2.2	57.3 ± 1.9	0.054
Pleomorphism (%)	35.3 ± 5.4	36.8 ± 4.0	0.376
Polymegathism (%)	48.7 ± 4.5	46.3 ± 3.4	0.086

^a^Independent samples t-test.

### Dendritic cells

There was a significant increase in the central corneal DC density (38.3 [17.5–97.3] vs. 13.5 [0–29.4] cells/mm^2^, *P* = 0.004) in subjects with Fabry disease compared to healthy control subjects (Table 2, Fig. 2).

### Male vs. Female participants

Male subjects with Fabry disease had a significantly lower NFD (19.7 ± 6.2 vs. 35.9 ± 5.7 fibers/mm^2^, *P* < 0.001) and NFL (13.7 ± 2.2 vs. 19.2 ± 1.8 mm/mm^2^, *P* < 0.001) compared with female subjects, whereas corneal sensitivity (*P* = 0.070), NBD (*P* = 0.193) and DC density (*P* = 0.887) did not differ. There were no significant differences in any of the study parameters among male and female participants in the control group (data not shown, *P* > 0.05 for all).

### Effect of enzyme replacement therapy

NFD was significantly lower (22.3 ± 8.8 vs. 33.9 ± 7.9 fibers/mm^2^, *P* = 0.018) in subjects receiving ERT (9 male, 2 female) compared to subjects not receiving ERT (1 male, 5 female), while corneal sensitivity (*P* = 0.301), NFL (*P* = 0.065), NBD (*P* = 0.884) and DC density (*P* = 0.591) did not show a significant difference (data not shown, *P* > 0.05 for all).

### Cornea verticillata

**Figure 3:**
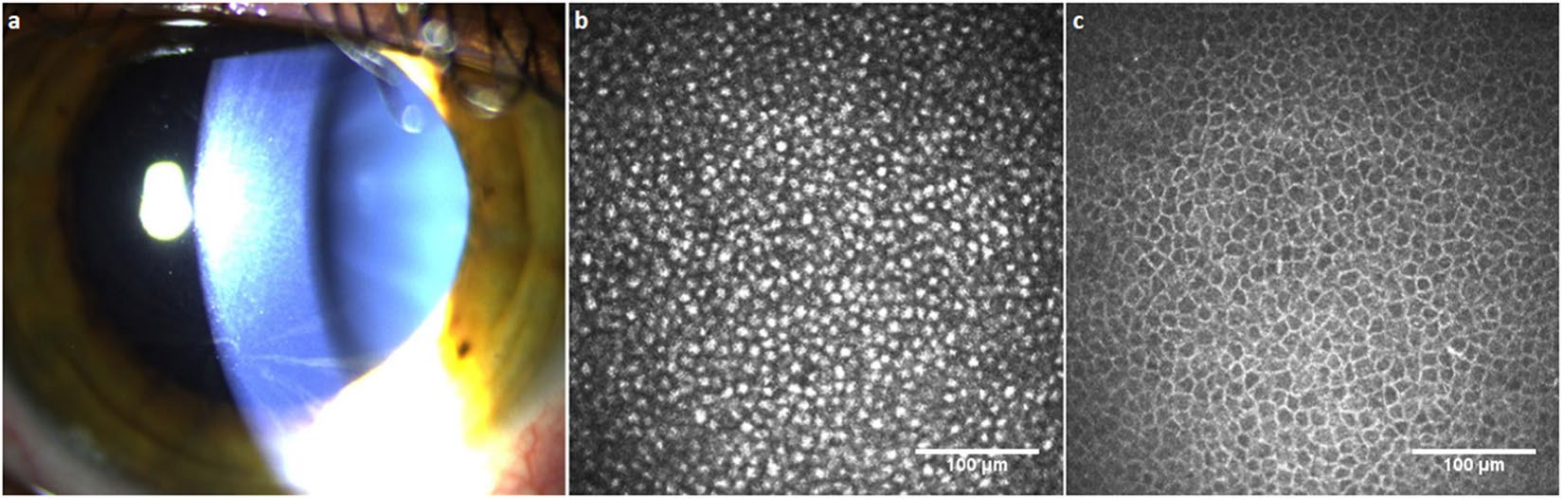
Slit-lamp biomicroscopic image showing cornea verticillata in a subject with Fabry disease (**a**). Corneal confocal microscopic images of the central cornea; showing hyperreflective inclusions in the basal epithelial cell layer of a subject with Fabry disease (**b**), and basal epithelial cells of a healthy control subject (**c**) with bright cell borders and dark cytoplasm.

Thirteen subjects with Fabry disease (8 male, 5 female; 76.5%) showed cornea verticillata on slit-lamp examination and hyperreflective intracellular inclusions in basal epithelial cells (Fig. 3). There were no significant differences in the Mainz Severity Score Index (MSSI), corneal sensitivity and corneal confocal microscopic measures between subjects with and without cornea verticillata (data not shown, *P* > 0.05 for all).

### Correlations

**Figure 4:**
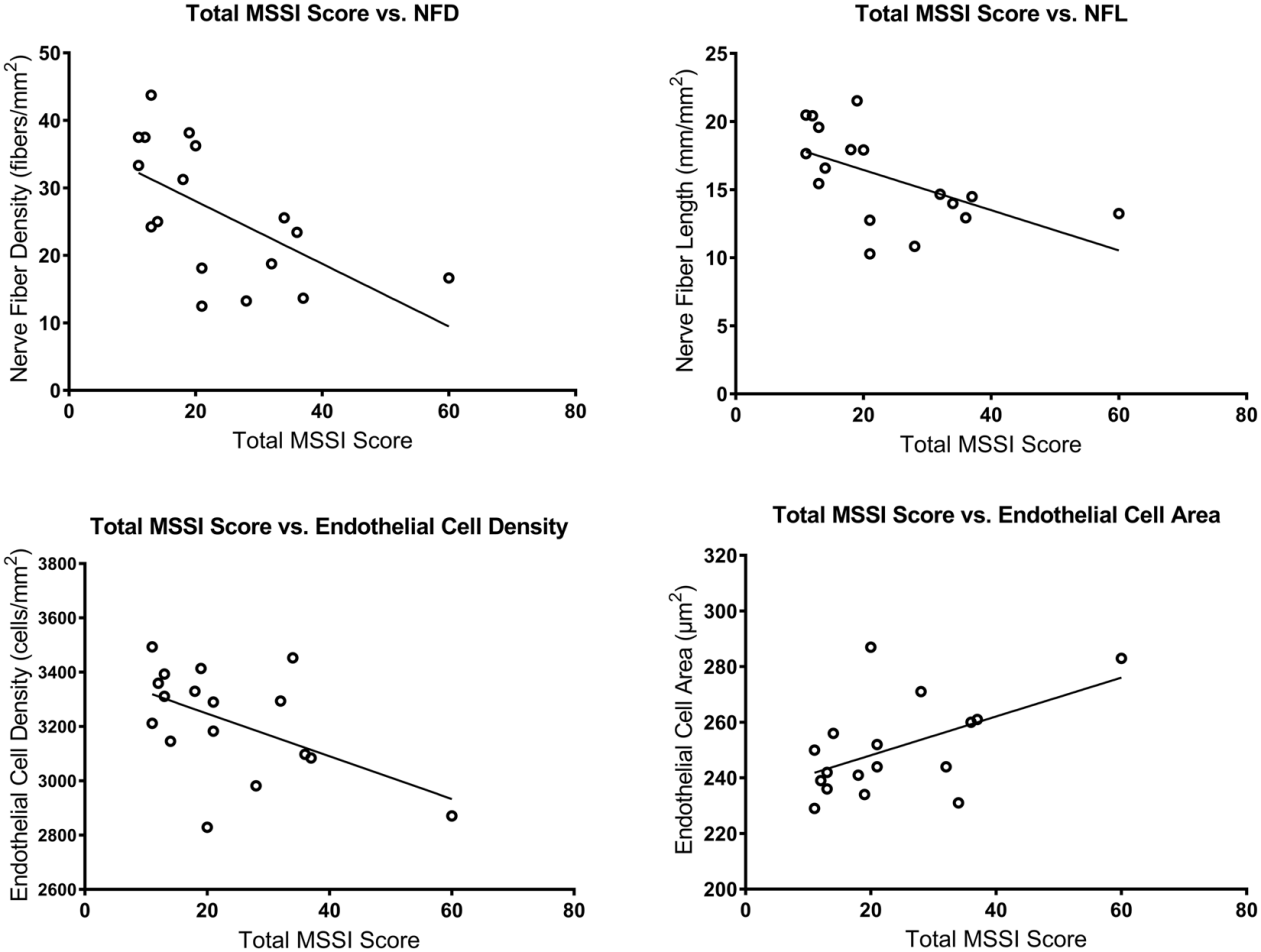
Scatter-plot graphs of correlations between the total Mainz Severity Score Index (MSSI) and nerve fiber density, nerve fiber length, endothelial cell density and endothelial cell area in subjects with Fabry disease.

**Figure 5:**
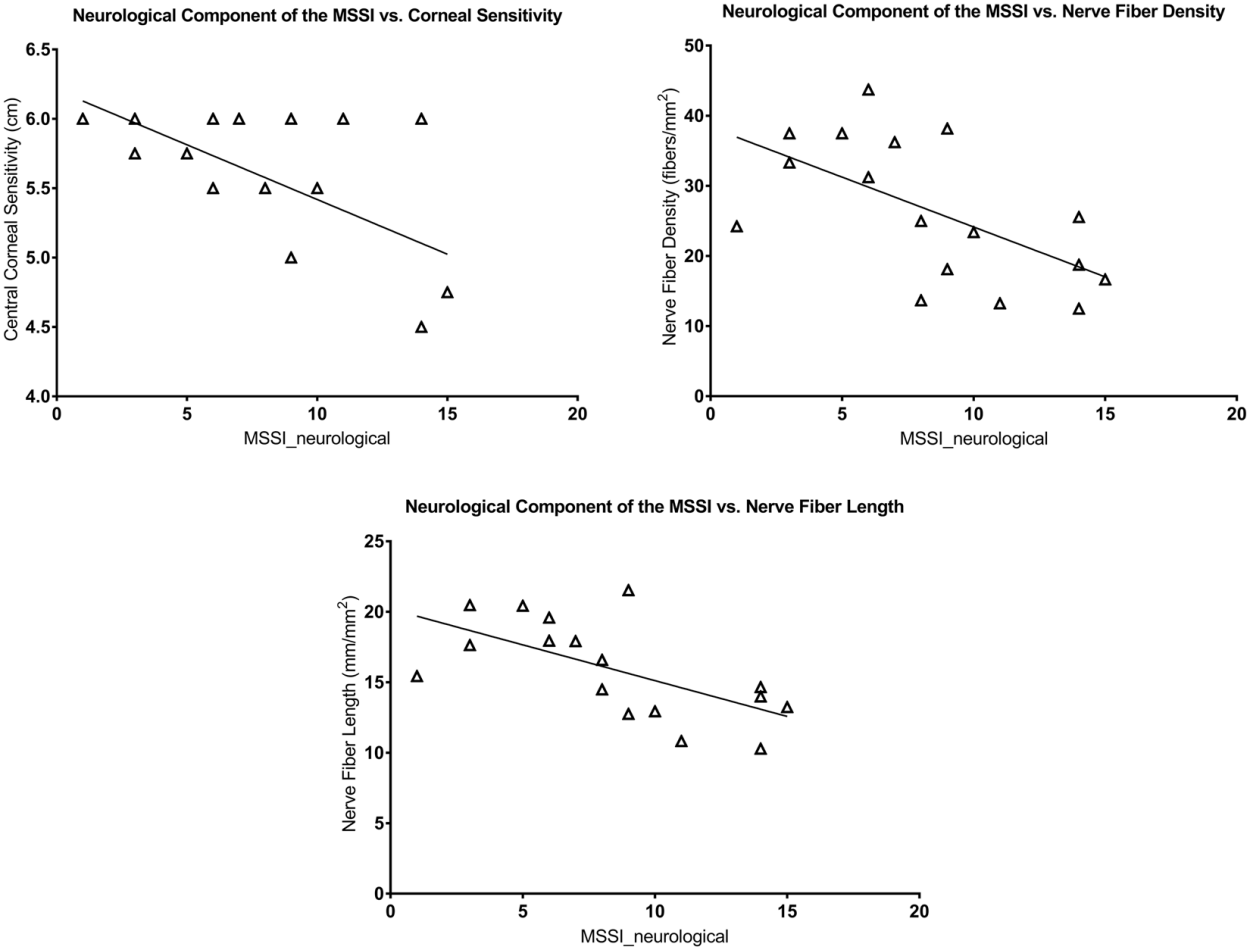
Scatter-plot graphs of correlations between the neurological component of the Mainz Severity Score Index (MSSI) and central corneal sensitivity, nerve fiber density and nerve fiber length in subjects with Fabry disease.

**Figure 6:**
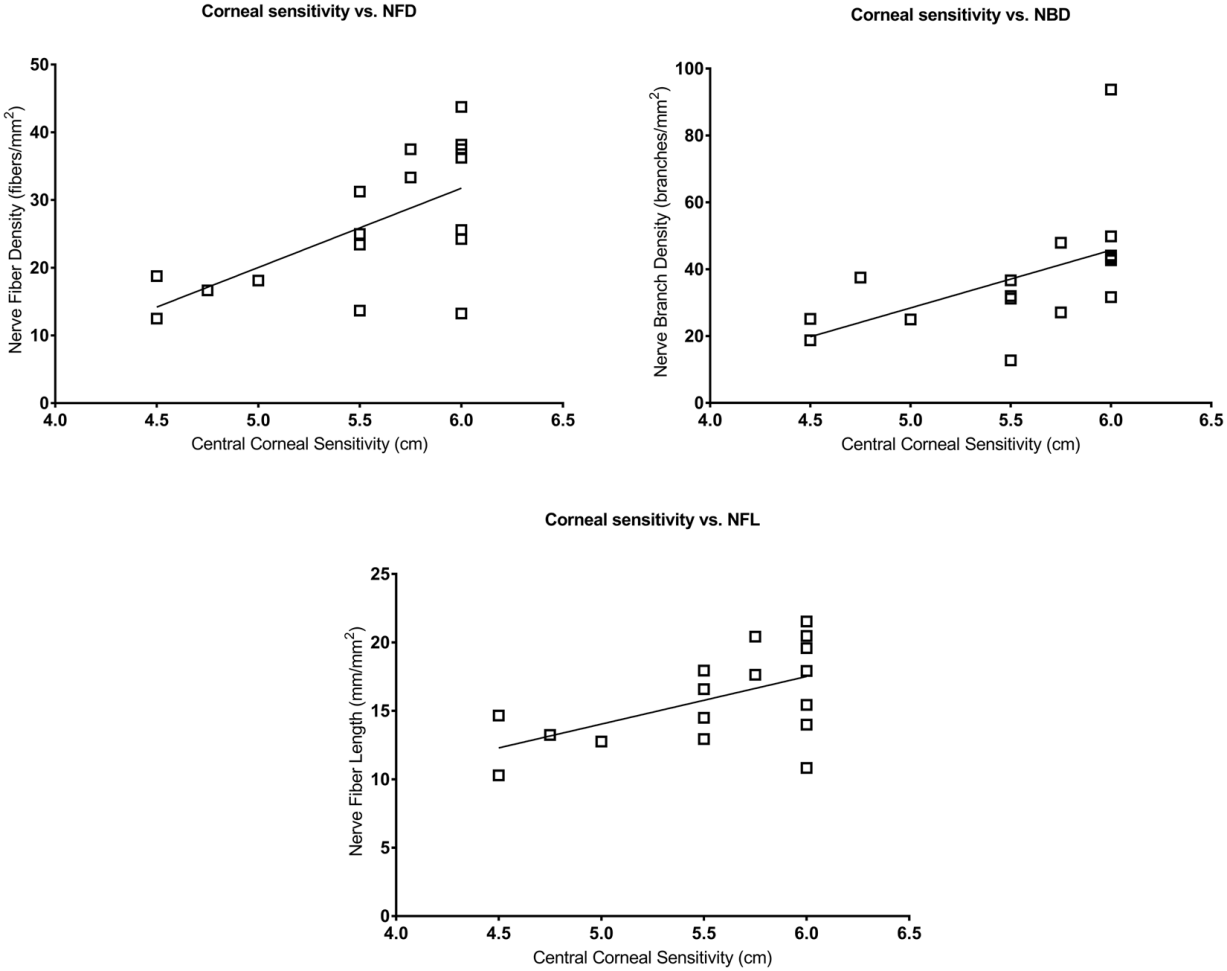
Scatter-plot graphs of correlations between central corneal sensitivity thresholds and nerve fiber density, nerve branch density and nerve fiber length in subjects with Fabry disease.

**Figure 7:**
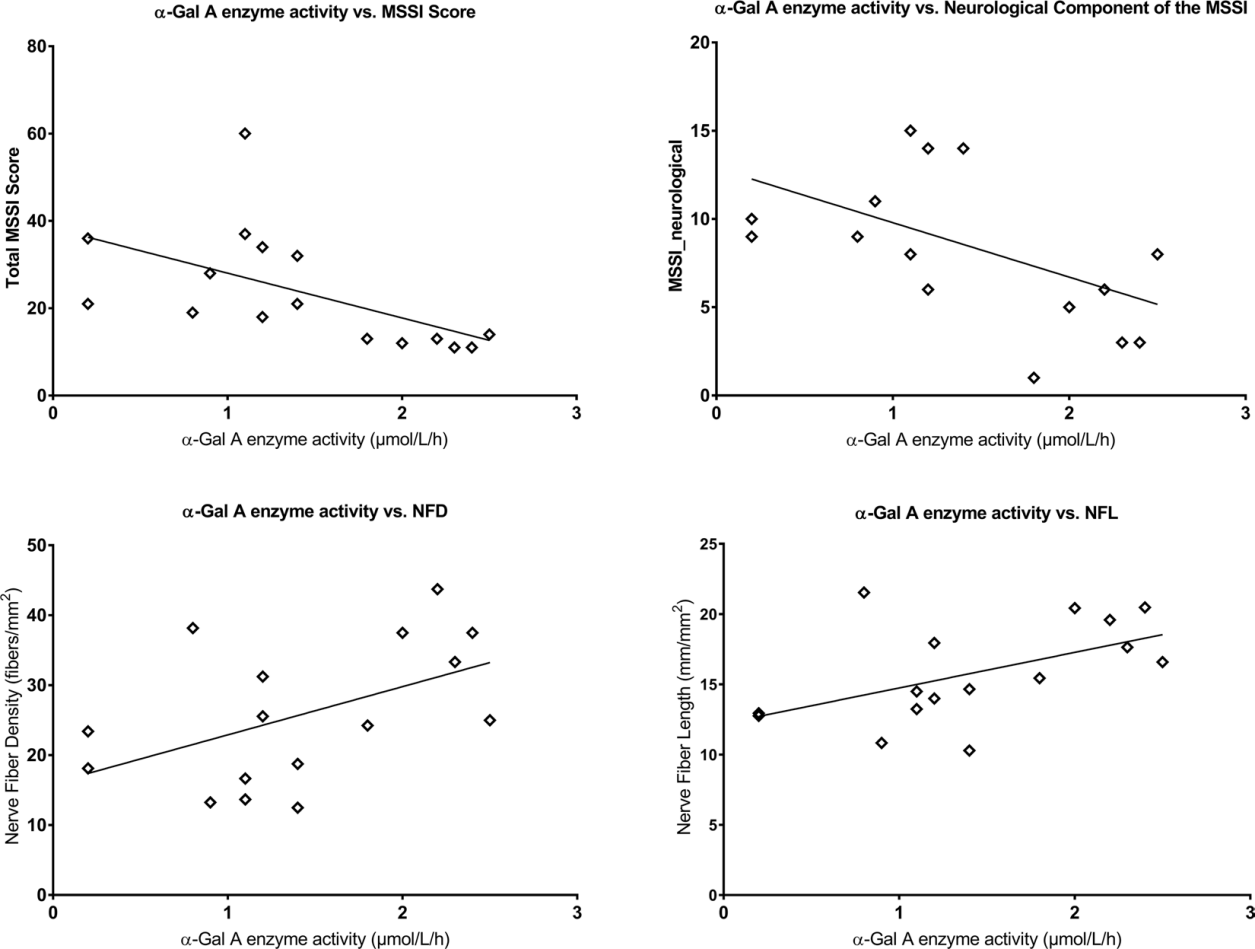
Scatter-plot graphs of correlations between α-galactosidase A enzyme activity and the total Mainz Severity Score Index (MSSI), the neurological component of the MSSI, nerve fiber density and nerve fiber length in subjects with Fabry disease.

The total MSSI score correlated with NFD (ρ = −0.686; *P* = 0.006), NFL (ρ = −0.692; *P* = 0.006), endothelial cell density (ρ = −0.511; *P* = 0.036) and endothelial cell area (ρ = 0.514; *P* = 0.036) (Fig. 4). The neurological component of the MSSI correlated with central corneal sensitivity (ρ = −0.489; *P* = 0.046), NFD (r = −0.598; *P* = 0.022) and NFL (r = −0.626; *P* = 0.021) (Fig. 5). Central corneal sensitivity correlated with NFD (ρ = 0.613; *P* = 0.018), NBD (ρ = 0.717; *P* = 0.003) and NFL (ρ = 0.500; *P* = 0.041) (Fig. 6). There were significant correlations of the α-galactosidase A enzyme activity with the total MSSI score (ρ = −0.723; *P* = 0.008), the neurological component of the MSSI (r = −0.515; *P* = 0.049), NFD (r = 0.498; *P* = 0.049) and NFL (r = 0.525; *P* = 0.049) (Fig. 7).

## Discussion

Neuropathic pain has been reported to be the most common presenting symptom of Fabry disease^4,5^, and has been related to loss of small fibers^33^. Quantification of nerve damage in subjects with Fabry disease is important for the initial diagnosis and assessment of neuropathy progression. Quantitative sensory testing has revealed small fiber dysfunction with a predominant abnormality for cold sensation in Fabry disease^8,33^. Marked reduction of small myelinated and unmyelinated nerve fibers has been demonstrated in histopathological studies of sural nerve biopsy specimens^7,34^. Additionally, reduced intraepidermal nerve fiber densities were found in up to 95% of subjects with Fabry disease^35,36^. In a detailed phenotyping study, warm and cold perception thresholds were elevated and the amplitude of pain related evoked potentials and intraepidermal nerve fiber density were significantly reduced in male but not female subjects with Fabry disease^37^. Whilst, sural nerve and skin biopsies allow objective quantification of small nerve fiber damage, they are invasive procedures which cannot be used for routine diagnosis and follow-up.

We and others have utilised corneal confocal microscopy to detect corneal nerve loss in a range of peripheral neuropathies^11–15^. Using a first generation CCM we previously reported significant corneal nerve loss in subjects with Fabry disease^25^. In the present study, we have found a significant reduction in corneal sensitivity and corneal nerve parameters, which were associated with each other and with disease severity assessed by MSSI as well as α-galactosidase A enzyme activity. Previous studies have reported more severe symptoms, neurological deficits and CCM abnormalities in hemizygous males compared to heterozygous females^4,25^. We have also found a significantly lower NFD and NFL in males compared to females, which could be explained by the heterogeneity in disease severity among females due to X-chromosome inactivation^4^. Additionally, we demonstrate an inverse correlation between the MSSI score and endothelial cell density which suggests that corneal endothelial cell abnormality is associated with the disease severity in subjects with Fabry disease.

In the present study whilst there was a trend for a reduction in NBD, this was not significant, unlike our previous study in Fabry disease^25^. We would attribute this to improved detection of corneal nerve branches due to the better resolution of the HRT III CCM used in this study.

In relation to underlying mechanisms of nerve damage, this is the first study to report increased DCs in the central cornea of subjects with Fabry disease. DCs have been shown to migrate and accumulate in the central cornea in various neuropathies and during inflammation^22,23,38,39^. Biancini *et al*.^31^ have reported that the inflammatory cytokines, IL-6 and TNF-α, are significantly increased in Fabry disease, indicating a pro-inflammatory state in these subjects. There are also reports proposing that Fabry disease has an autoimmune component in its pathophysiology^40–42^. A flow cytometry study has shown a reduction in circulating DCs in peripheral blood samples, suggestive of increased extravasation and migration to peripheral tissues^30^. The increased DC density in the central cornea of subjects with Fabry disease may reflect this process and therefore CCM may provide further insight into the role of immune mechanisms in Fabry disease.

The reported prevalence of cornea verticillata was 76.9% in heterozygous females and 73.1% in hemizygous males according to the Fabry Outcome Survey^43^. Hyperreflective intracellular inclusions in the basal epithelium have been found using CCM in subjects with Fabry disease^44,45^. In this study, whilst cornea verticillata was observed in 71.4% of females and 80.0% of males, there was no difference in the MSSI score, corneal sensitivity or corneal confocal microscopic measures between subjects with and without cornea verticillata. This is in agreement with previous reports suggesting that the presence of cornea verticillata is not associated with disease severity, or the systemic, renal and cardiac manifestations of the disease^43,46^.

In relation to the effects of treatment with ERT, Schiffmann *et al*. reported an improvement in the neuropathic pain severity score^47^ and small nerve fiber function^48^. Whilst one study showed no change in intraepidermal nerve fiber density after 12–18 months of treatment with ERT^10^, another study did show an improvement in proximal intraepidermal nerve fiber density after 4 years of ERT^33^. In our study, despite treatment with ERT, the NFD was lower compared to subjects not on ERT. However, this may be attributed to the fact that in clinical practice only the more severely affected subjects are commenced on ERT.

A limitation of this study is the acquisition and selection of the CCM images by an unmasked observer, which may increase the risk of bias^49^. However, corneal nerve quantification was undertaken using automated software, and hence the analysis was not operator-dependent. The use of a standardized image selection protocol, as used in this study, has also been shown to have excellent intra- and inter-observer repeatability^50^. Other limitations are the small sample size which has not allowed us to undertake further subgroup analysis. The cross-sectional nature of the study design also precludes the ability to draw causal inferences. However, we believe that our data show that CCM could have considerable utility in the non-invasive quantification of neural and immune pathology in Fabry disease. Longitudinal studies are required to evaluate the utility of CCM for monitoring changes in corneal nerve morphology and DC density, especially in relation to the effects of ERT or other therapeutic strategies.

## Methods

This cross-sectional comparative study was undertaken at a tertiary referral university hospital between February 2017 and November 2017. The study design fulfilled the tenets of the Declaration of Helsinki and was approved by the Clinical Research Ethics Committee of the Necmettin Erbakan University (2017/805). Written informed consent was obtained from all participants after a detailed explanation of the nature and possible consequences of the study.

The diagnosis of Fabry disease had been previously established on the basis of clinical features, biochemical evidence of reduced α-galactosidase A enzyme activity and molecular analysis of the GLA gene. α-Galactosidase A enzyme activity was assessed in dried blood spots and GLA gene mutation analysis was performed by PCR amplification and direct automatic sequencing, as we have previously described in detail^3^. All consecutive subjects with Fabry disease who were eligible for CCM analysis were enrolled in the study. Control participants were individuals without any systemic or ocular disease. Exclusion criteria were previous ocular trauma or surgery, any corneal pathology, contact lens use, and any other systemic disease that might affect the cornea. None of the participants were receiving anti-inflammatory or immuno-modulatory medications. All subjects underwent clinical and ophthalmological examination, and disease severity was assessed using the MSSI score ranging from 0–76 which includes scoring for general (0–18), neurological (0–20), cardiovascular (0–20), and renal (0–18) symptoms and signs of Fabry disease^51^.

### Corneal Sensitivity Assessment

Central corneal sensitivity was measured using a contact corneal esthesiometer (Cochet-Bonnet; Luneau, France). The esthesiometer is based upon the principle of pressure transmitted axially by a 0.12 mm-diameter nylon monofilament, which was applied with a low pressure perpendicular to the center of the cornea. The filament length was progressively reduced from 6 cm in 5-mm steps until the first response occurred. The longest filament length (cm) resulting in a positive response was verified twice and recorded as a measure of central corneal sensitivity. Both eyes of the study participants were evaluated but only the data obtained form the right eye were included in statistical analyses.

### Corneal Confocal Microscopy

All participants underwent examination with a laser scanning CCM using the Rostock Corneal Module/Heidelberg Retina Tomograph lll (Heidelberg Engineering, Germany). The full thickness of the central cornea was scanned using the “section” mode. A standardized image selection protocol was used^50^. Three high-quality nerve plexus images containing the highest, intermediate and least number of nerve fibers were selected from each subject and the average of these results was considered. Automated CCMetrics software, ver. 2.0 (University of Manchester, UK) was used to quantify the nerve fibers^52^. Three parameters were quantified: corneal nerve fiber density (NFD), the total number of major nerves/mm^2^; nerve branch density (NBD), the number of branches emanating from major nerve trunks/mm^2^; nerve fiber length (NFL), the total length of all nerve fibers and branches (mm/mm^2^). The same image frames were used to quantify DC density. The number of highly reflective cells with dendriform structures was counted manually and the density was derived as the number of cells in the area of frame assessed in square millimeters (number/mm^2^) using the proprietary software within the corneal confocal microscope. Corneal endothelial cell morphology was analysed using the Corneal Endothelium Analysis System (CEAS) software (University of Bradford, UK)^53^ and five parameters were quantified: endothelial cell density (cells/mm^2^), endothelial cell area (µm^2^), endothelial cell perimeter (µm), pleomorphism (percentage of hexagonality coefficient, calculated as the number of hexagonal-shaped endothelial cells divided by the total number of cells) and polymegathism (percentage of coefficient of variation in cell size, calculated as the standard deviation of cell area divided by the mean endothelial cell area). For all subjects, only the right eye was included in analyses. All image acquisition and analysis were performed by a single unmasked observer (GB).

### Statistical Analysis

Statistical analysis was performed using SPSS ver. 21.0 (Chicago, IL, USA) software. Basic descriptive statistics were calculated on all the data and are reported as the mean ± SD or median (interquartile range [IQR]), as appropriate. The Pearson *χ*^2^-test was used to compare categorical variables. Normal distribution of continuous variables was confirmed with the Kolmogorov-Smirnov test. Independent samples t-test for normally distributed data and Mann-Whitney U-test for non-normally distributed data were used to compare the parameters between the subjects with Fabry disease and healthy control participants. The associations between disease severity scores, corneal sensitivity and confocal microscopic parameters were measured using Pearson’s correlation coefficient for normally distributed data and Spearman’s correlation coefficient for non-normally distributed data. A Benjamini-Hochberg correction was applied to adjust the *P* values for multiple correlations. For all evaluations, a *P* value of less than 0.05 was considered statistically significant.

## Electronic supplementary material

Supplementary Table S1

## Data Availability

The datasets generated during and/or analysed during the current study are available from the corresponding author on reasonable request.
