# Supplementary material for: Corneal confocal microscopy detects corneal nerve damage and increased dendritic cells in Fabry disease
Source: Sci Rep. 2018 Aug 16;8:12244. doi: 10.1038/s41598-018-30688-z (PMC6095897; doi:10.1038/s41598-018-30688-z)
Supplement: Supplementary file 1 — Supplementary Table S1 [file 41598_2018_30688_MOESM1_ESM.pdf]

Corneal confocal microscopy detects corneal nerve damage and increased dendritic cells in Fabry disease

Gulfidan Bitirgen[1*]

Kultigin Turkmen[2]

Rayaz A. Malik[3,4]

Ahmet Ozkagnici[1]

Nazmi Zengin[1]

1. Department of Ophthalmology, Necmettin Erbakan University Meram Faculty of Medicine, Konya, Turkey

2. Division of Nephrology, Department of Internal Medicine, Necmettin Erbakan University Meram Faculty of Medicine, Konya, Turkey

3. Weill Cornell Medicine-Qatar, Education City, Doha, Qatar

4. Central Manchester University Teaching Hospitals Foundation Trust and Division of Cardiovascular Sciences, University of Manchester, Manchester, UK

Supplementary Table S1. Comparison of the central corneal sensitivity, corneal nerve fiber and endothelial cell parameters and dendritic cell density among the subgroups of different gene mutation types in subjects with Fabry disease.

	p.N34H mutation (n=13)	p.Leu54Phe mutation (n=3)	*P* value
Central corneal sensitivity (cm, median [IQR])	5.75 [5.50 - 6.00]	5.50 [5.00 - 6.00]	0.611[a]
Nerve fiber density (fibers/mm^2, mean ± SD)	27.1 ± 10.4	26.6 ± 10.4	0.935[b]
Nerve branch density (branches/mm^2, median [IQR])	36.7 [29.2 - 43.9]	25.0 [12.7 - 93.7]	0.521[a]
Nerve fiber length (mm/mm^2, mean ± SD)	16.2 ± 3.3	15.7 ± 5.0	0.854[b]
Dendritic cell density (cells/mm^2, median [IQR])	38.3 [26.8 - 103.4]	13.5 [0 - 38.2]	0.146[a]
Endothelial cell density (cells/mm^2, mean ± SD)	3244.3 ± 190.3	3231.7 ± 163.5	0.917[b]
Endothelial cell area (μm^2, mean ± SD)	248.5 ± 16.5	248.7 ± 13.3	0.990[b]
Endothelial cell perimeter (μm, mean ± SD)	57.1 ± 2.0	57.3 ± 1.2	0.838[b]
Pleomorphism (%)	36.5 ± 4.4	36.3 ± 2.5	0.939[b]
Polymegathism (%)	46.8 ± 3.7	45.0 ± 2.6	0.447[b]

[a]Mann-Whitney test. [b]Independent samples t-test.
